# Emerging Trend in the Pharmacotherapy of Osteoarthritis

**DOI:** 10.3389/fendo.2019.00431

**Published:** 2019-07-02

**Authors:** Wei Zhang, William Brett Robertson, Jinmin Zhao, Weiwei Chen, Jiake Xu

**Affiliations:** ^1^School of Medicine, Southeast University, Nanjing, China; ^2^Department of Integrative Medical Biology, Umeå University, Umeå, Sweden; ^3^Australian Institute of Robotic Orthopaedics, Perth, WA, Australia; ^4^School of Surgery, The University of Western Australia, Perth, WA, Australia; ^5^School of Science, Faculty of Science and Engineering, Curtin University, Perth, WA, Australia; ^6^Department of Materials Science and Engineering, College of Engineering, University of North Texas, Denton, TX, United States; ^7^Research Centre for Regenerative Medicine, Guangxi Medical University, Nanning, China; ^8^Department of Orthopaedic Surgery, The First Affiliated Hospital of Guangxi Medical University, Nanning, China; ^9^Guangxi Key Laboratory of Regenerative Medicine, Guangxi Medical University, Nanning, China; ^10^School of Biomedical Sciences, The University of Western Australia, Perth, WA, Australia

**Keywords:** osteoarthritis, articular cartilage, clinical trials, pharmacologic therapy, regenerative therapy

## Abstract

Osteoarthritis (OA) is a degenerative joint disorder and one of the most prevalent diseases among the elderly population. Due to the limited spontaneous healing capacity of articular cartilage, it still remains challenging to find satisfactory treatment for OA. This review covers the emerging trends of pharmacologic therapies for OA such as traditional OA drugs (acetaminophen, non-steroidal anti-inflammatory drugs (NSAIDs), opioids, serotonin-norepinephrine reuptake inhibitors (SNRIs), intra-articular injections of corticosteroids, and dietary supplements), which are effective in pain relief but not in reversing damage, and are frequently associated with adverse events. Alternatively, disease-modifying drugs provide promising alternatives for the management of OA. The development of these emerging OA therapeutic agents requires a comprehensive understanding of the pathophysiology of OA progression. The process of cartilage anabolism/catabolism, subchondral bone remodeling and synovial inflammation are identified as potential targets. These emerging OA drugs such as bone morphogenetic protein-7 (BMP-7), fibroblast growth factor-18 (FGF-18), human serum albumin (HSA), interleukin-1 (IL-1) inhibitor, β-Nerve growth factor (β-NGF) antibody, matrix extracellular phosphoglycoprotein (MEPE) and inverse agonist of retinoic acid-related orphan receptor alpha (RORα) etc. have shown potential to modify progression of OA with minimal adverse effects. However, large-scale randomized controlled trials (RCTs) are needed to investigate the safety and efficacy before translation from bench to bedside.

## Introduction

Osteoarthritis (OA) is one of the most prevalent diseases among the elderly population. In the USA, symptomatic knee OA occurs mostly in people aged 60 and older with an overall rate of 10% of men and 13% of women (1). OA often severely affects patients' quality of life and symptoms include pain, stiffness, swelling, tenderness, and loss of joint range of motion (2). The main cause of OA is the “wear and tear” of cartilage over time, which has been identified with limited self-repair capacity.

Currently, there is no cure for OA. Existing treatments aim to reduce pain and symptoms, as well as improve joint functional capacity (3). According to the guidelines from the American Academy of Orthopaedic Surgeons (AAOS) and the Osteoarthritis Research Society International (OARSI), treatment options include exercise, weight management, physical therapy, medications, and surgery (3, 4). Appropriate exercises help strengthen muscles and improve joint function (5). Weight loss is encouraged to those who are overweight (6). Assistive devices, such as canes and walkers are recommended for patients with symptomatic knee OA (3). Complementary and alternative medicine such as nutritional supplements, massage, and topical acupuncture have been indicated but more clinical data is required to support their efficacy (7). Painkiller and anti-inflammation medication are the most commonly used treatments. However, they are only helpful for relieving OA symptoms and are sometimes associated with adverse side effects (8). Surgery is only considered for those who exhibit severe symptoms and are unresponsive to conservative therapy. Arthroscopic irrigation and debridement, drilling, and microfracture techniques show short-term effects, but are not beneficial in the long-term (9). Total joint replacement is clinically effective and cost-effective intervention for patients with advanced OA. However, it is not suitable for young patients due to its finite lifespan (10–15 years) (10).

With the better understanding of the pathophysiology of OA progression, it has been recognized that OA involves the whole joint, rather than the cartilage itself. Emerging disease-modifying drugs hold promise for OA management by regulating cartilage anabolism/catabolism, subchondral bone remodeling or synovial inflammation. This review will focus on current pharmacologic therapy for OA patients. It covers the traditional medicine that has been used in practice for decades and explores the emerging medications with disease-modifying drugs which have shown promise in clinical and preclinical studies (Table 1). The indications, mechanisms of action and side effects of each OA drug will be discussed.

**Table 1:** Pharmacotherapeutic options for osteoarthritis treatment.

**Classification**	**Name**	**Indications**	**Pharmacological mechanisms**	**Side effects**	**References**
Traditional medications	Acetaminophen	Relief mild to moderate pain relief	Inhibit COX-3 activity and synthesizing prostaglandin.	Liver damage; liver toxicity; transient liver enzyme elevations and hepatotoxicity	(3, 4, 11–15)
	Non-steroidal anti-inflammatory drugs (NSAIDs)	Relief mild to moderate pain; analgesic and anti-inflammatory	Inhibit cyclooxygenase enzymes and prostaglandin synthesis; inhibit COX-1 and COX-2 activity	Gastrointestinal complications, Kidney disease and adverse cardiovascular events	(13, 16–22)
	Opioid analgesics	Pain relief	Inhibit pain pathways in the central nervous system	Nausea, vomiting, headache, constipation, fatigue and drowsiness	(3, 18, 23–27)
	Serotonin-norepinephrine reuptake inhibitors (SNRIs)	Treatment of depression and mood disorder	Inhibit serotonin-norepinephrine reuptake	Fatigue and somnolence; sexual dysfunction; gastrointestinal problems	(28–32)
	Intra-articular injections of corticosteroids	Relief moderate-to-severe pain and inflammation	Down-regulate genetic expression of pro-inflammatory proteins; decrease inflammatory markers and cytokines	Post-injection pain and flushing; septic arthritis; possible rare Tachon syndrome	(33–38)
	Vitamin D supplements	Reduction of WOMAC pain and WOMAC function; reduction of VAS pain	Increase calcium absorption and have effects on cartilage and bone metabolism	No severe safety issues were reported	(39–42)
	Glucosamine and chondroitin sulfate supplements	Management of the symptoms of OA	Inhibit catabolic enzymes activities, and reduce IL-1 β levels in synovial fluids	No severe safety issues were reported	(15, 43–48)
	Antioxidant supplements	Pain relief and function improvement in knee OA	Inhibit reactive oxygen species signal transduction	Large-scale RCTs are needed	(49–52)
Emerging medications	Bone morphogenetic protein-7 (BMP-7)	Limit progression of osteoarthritis; treat bone nonunion and spinal fusion	Promote embryogenesis, improve bone and cartilage formation and repair	No adverse effects were found	(53–56)
	Fibroblast growth factor-18 (FGF-18)	Target cartilage for knee OA	Promote chondrogenesis and cartilage repair; increase cartilage thickness	No severe safety issues were reported	(57–59)
	Human serum albumin (HSA)	Improvement of regeneration of cartilage and pain relief in knee OA; anti-inflammatory for severe knee OA	Inhibit the production of pro-inflammatory cytokine in T-cells; induce transcriptional changes of mesenchymal stem cell; up-regulate COX-2, prostaglandin E2 and D2	No serious drug-related AEs	(17, 60–63)
	β-Nerve growth factor (β-NGF) antibody	Modulation of chronic pain	Have effects on nociceptor sensitization	Headache, upper respiratory tract infection and paresthesia	(64–71)
	Interleukin-1 (IL-1) inhibitor	Anti-inflammation	Inhibit the synthesis of proteolytic enzymes, pro-inflammatory cytokines and the degradation of cartilage extracellular matrix	No data available	(72–77)
	Matrix extracellular phosphoglycoprotein (MEPE)	Target subchondral bone remodeling	Regulate subchondral bone mineralization	No serious drug-related AEs	(78, 79)
	Parathyroid hormone (PTH)/ Parathyroid hormone-related protein (PTHrP)	Regulate subchondral bone remodeling and terminal differentiation of articular cartilage	Inhibit chondrocyte hypertrophy, terminal differentiation and degradation	No data available	(80–83)
	Transforming growth factor β (TGF-β) inhibitor	Target subchondral bone remodeling	Inhibit subchondral bone pathology in response to altered mechanical loading	No data available	(84)
	Cholesterol metabolism and inverse agonist of retinoic acid-related orphan receptor alpha (RORα)	Target CH25H-CYP7B1 cholesterol metabolism axis	reduce cartilage destruction and inhibit the up-regulation of MMP3 and MMP13	No data available	(85, 86)

## Traditional Medications

The traditional medications can be categorized into acetaminophen, non-steroidal anti-inflammatory drugs (NSAIDs), opioid analgesics, serotonin-norepinephrine reuptake inhibitors (SNRIs), intra-articular (IA) injections of corticosteroids, and dietary supplements. These drugs are well-documented in the literature and are commonly used in today's clinical management of OA. Although there are generally accepted guidelines for these traditional drugs, they need to be used with caution due to the growing concerns with adverse effects.

### Acetaminophen

Acetaminophen, commonly called paracetamol, is an over-the-counter (OTC) analgesic, which is recommended by all guidelines as the first line treatment for people with mild to moderate OA. Acetaminophen has weak effects on inflammatory factors cyclooxygenase (COX)-1 and COX-2, which are essential to the synthesis of prostaglandins (PGs) (87). Acetaminophen produces potent inhibitory actions for COX-3 to decrease pain and fever (11, 12). Results from 7 randomized controlled trials (RCTs) comparing acetaminophen to placebo showed that acetaminophen was superior to placebo on overall pain reduction with statistical significance (*p* < 0.05) but not on Western Ontario and McMaster Universities Osteoarthritis Index (WOMAC) and Lequesne outcomes (13). No significant differences in safety profile between acetaminophen and placebo were detected in these RCTs. Although acetaminophen is regarded as the first line OA therapies, it is reported that taking acetaminophen over the recommended dosage can cause liver damage or liver failure. According to ACR and OARSI guidelines, 4,000 mg per day is the maximum recommended therapeutic dosage and appears to be an effective initial treatment for mild to moderate knee or hip OA (3, 14, 15). AAOS guideline further reduced the amount of acetaminophen to no more than 3,000 mg per day to minimize its risk of liver damage (4). Therefore, acetaminophen is a mild and relatively safe analgesic but should be used on a strict regimen according to the recommended dosage and long-term use is to be avoided to lower the risk of reported side effects.

### NSAIDs

NSAIDs are often used for patients with symptomatic hip or knee OA and are non-responsive to acetaminophen. This family of drugs provide the suppression of cyclooxygenase enzymes activity and the resulting inhibition of PGs, especially COX-1 and COX-2, and have analgesic, antipyretic and anti-inflammatory effects (16). The efficacy of NSAIDs for the treatment of OA is well-documented. It has been reported to show an overall improvement over acetaminophen on pain relief from meta-analysis studies of 8 RCTs (13, 17). However, health problems greatly limit their widespread use, and it has been suggested that it's use is limited to short-term use in the elderly with OA due to the high risk of NSAIDs-induced side effects, (18). Among people taking NSAIDs, the incidence of side effects is estimated to be about 30% (19). Gastrointestinal (GI) complications are one of the most common side effects, which happen to 1–2% people using NSAIDs every year, significantly higher than that of people who don't use NSAIDs (20, 21). Kidney disease and adverse cardiovascular events are also reported to be associated with NSAIDs use (22). Although selective COX-2 inhibitors, which suppress both COX-1 and COX-2 are proven safer than traditional NSAIDs, several marketed COX-2 inhibitors have been withdrawn by the Food and Drug Administration (FDA) due to their reported side effects in clinical use, such as Rofecoxib (Vioxx, Merck) and Valdecoxib (Bextra, Pfizer). Therefore, the benefit/risk ratio has become an important indicator when taking NSAIDs, and the minimum effective dose and short-term use are recommended.

### Opioid Analgesics

Only when the first line therapies, such as NSAIDs or acetaminophen, are ineffective or contraindicated, are weak opioids and narcotic analgesics used to control refractory pain in patients with hip or knee OA (3). The mechanism by which opioids act on OA is to inhibit the pain pathway in the central nervous system through binding the mu opioid receptor (18). Compared with placebo, significant lower pain intensity and small benefits on physical function were associated with the use of opioids (23). However, the relatively high risk of side effects including nausea, vomiting, dizziness, constipation, drowsiness, fatigue, and headache limit opioid usefulness (18, 24, 25). From a meta-analysis of 18 randomized placebo-controlled trials, 25% (818/3244) of patients in the opioid group withdrew from studies, which is much higher than 7% (116/1612) in the placebo group (23). Another problem with opioid analgesics is the risk of overdose deaths or addiction (26). It is reported that opioid overdose deaths increased significantly in the U.S, a 14% increase from 2013 to 2014 (27). Opioid addiction also increased at the same time with approximately 2.5 million adults affected in 2014 (26). Therefore, long-term use is not recommended due to the side effects and the addictive risk of opioid analgesics.

### SNRIs

SNRIs are mainly used to treat depression and other mood disorders by inhibiting serotonin-norepinephrine reuptake (28). Duloxetine, a selective SNRI was approved by the FDA for clinical treatment of chronic musculoskeletal pain including OA. As serotonin and norepinephrine imbalance in the central pain pathway is associated with central sensitization and chronic pain, duloxetine is effective to treat chronic OA pain resulting from the dysfunction of central pain pathways (29). This class of drug is conditionally recommended in patients who had an inadequate response to initial therapies such as acetaminophen and NSAIDs. When compared with placebo-control, duloxetine showed significantly greater improvement on pain reduction and physical function in 2 double-blinded RCTs (30, 31). However, from the 2012 Cochrane review regarding doxycycline for OA of the knee or hip, the authors concluded that doxycycline is not associated with clinical benefits in pain relief or joint function, while the small benefit related to decreasing joint space narrowing might have little clinical relevance considering their potential side effects (32). The most common side effects of SNRIs are gastrointestinal problems, sexual dysfunction, fatigue and somnolence (28). More large-scale RCTs are needed to investigate the safety and efficacy of SNRIs for OA treatment.

### IA Injections of Corticosteroids

IA injections of corticosteroids are recommended for patients with moderate-to-severe pain and not responding to first line medication (3). The five FDA-approved injectable corticosteroids are methylprednisolone acetate, triamcinolone acetate, betamethasone acetate and betamethasone sodium phosphate, triamcinolone hexacetonide, and dexamethasone (88). The anti-inflammatory and immunosuppressive effect of IA corticosteroids is immediate and pronounced by acting directly on nuclear steroid receptors and impeding the inflammatory and immune cascade, and thus down-regulating the expression of pro-inflammatory proteins and decreasing inflammatory cytokines (33, 34, 88). From the latest version of Cochrane systematic review of intra-articular corticosteroid for knee OA, IA corticosteroids are indicative of beneficial effects in pain reduction and functional improvement than IA placebo based on 27 trials with 1,767 participants (35). However, most of the identified trials had a low quality of evidence and inconsistency between trials. Therefore, the benefits of IA injections of corticosteroids remain unclear, and further research is required. The reported side effects of IA injections of corticosteroids are mostly post-injection pain and flushing, septic arthritis, and some rare cases of Tachon Syndrome (36, 37). Chronic use of corticosteroids has been reported to induce osteoporosis in children with 21-hydroxylase deficiency (38).

### Vitamin D Supplements

Vitamin D deficiency is associated with development or progression of OA as vitamin D has effects on calcium absorption in cartilage and bone metabolism (39, 40). There are contradictory reports as to whether vitamin D supplements can reduce OA pain. A meta-analysis of 4 RCTs with 1136 patients revealed that vitamin D supplementation significantly decreased WOMAC pain and loss of function, but had no effect on WOMAC stiffness nor tibial cartilage volume (41). Another controlled before-after study of 175 patients with knee OA showed that receiving 40,000 IU vitamin D2 per week for 6 months resulted in reduction of visual analog scale (VAS) pain and improvement in the quality of life, grip strength and physical performance (40). However, in a 2-year NIH-funded RCTs of 146 knee OA patients receiving oral doses of cholecaliciferol to raise plasma levels of vitamin D, there was no reduction in knee pain or cartilage volume loss compared to placebo control (42). Thus, vitamin D supplementation appears to have a positive clinical effect on pain modification but conflicting results exist. Additional long-term clinical trials are required to further determine its clinical benefit.

### Glucosamine and Chondroitin Sulfate Supplements

Glucosamine is a major component of glycosaminoglycans in cartilage matrix. Glucosamine sulfate has been found to induce human chondrocytes to produce proteoglycans, and to suppress catabolic enzymes as well as IL-1β production in synovial fluids (43, 44). Chondroitin sulfate, a major constituent of the extracellular matrix in articular cartilage (45), plays an important role in regulating osmotic pressure and collagen network homeostasis (46). Over the past 20 years, glucosamine and chondroitin sulfate have been widely used for the management of the symptoms of OA (47, 48). A meta-analysis reported a delay of the radiological progression of OA of the knee after the treatment of glucosamine sulfate and chondroitin sulfate, in particular, if used in long-term (43). Glucosamine sulfate and chondroitin sulfate are comparable in short-term use, while glucosamine sulfate shows protective effects on minimum joint space narrowing in long-term use. However, ACR does not recommend glucosamine or chondroitin as the initial treatment of OA in the latest guideline and does not assure their consistency (15).

### Antioxidant Supplements

Reactive oxygen species (ROS) has been identified to be involved in the pathology of OA. It is reported that increased oxidative stress can be detrimental to the chondrocytes and free radicals can promote the senescence and apoptosis of chondrocytes by activating the NFκB, PI3K, and JNK pathways (49). Compared to the controls, the oxidant parameters (total peroxide, lipid hydroperoxide, and oxidative stress index) were significantly higher whereas the antioxidant parameters (serum thiol levels, thiol level, prolidase, and catalase activity) were lower in patients with grade II-III knee OA (50). Therefore, the application of antioxidant supplements may hold promise for the treatment of OA via the inhibition of ROS production. Although antioxidant supplements haven't got any approval from FDA for OA treatment, Vitamin supplements (vitamins A, E, and C), and natural herbs extract (turmeric, avocado, and boswellia) have been attempted for OA treatment, and the benefits of these antioxidant supplements have been confirmed with reduction of pain and improvement of join function (51, 52). It is advisable to consult doctors before taking these supplements to lower the potential side effects and avoid interactions with other OA medications.

## Emerging Medications

Due to the limitation of traditional OA drugs, the ongoing search for more effective OA drugs with satisfactory treatment effects (i.e., alleviating pain, relieving symptoms, and restoring the normal joint structure) and minimal side-effects is still necessary. A few emerging disease-modifying drugs have shown promise in modifying OA progression by regulating cartilage anabolism/catabolism, subchondral bone remodeling or synovial inflammation. These include growth factors, cytokines, monoclonal antibodies and inhibitors, which help reduce inflammation, promote chondrogenesis, and inhibit osteogenesis and matrix degradation.

### Bone Morphogenetic Protein-7 (BMP-7)

BMP-7 (also known as OP-1), a member of the transforming growth factor (TGF)-β superfamily has been approved by FDA as a biologic for bone non-union and spinal fusion therapy (53). It plays key roles in embryogenesis and bone and cartilage homeostasis and regeneration (54). In articular cartilage, BMP-7 has anabolic effects by increasing the synthesis of cartilage-specific extracellular matrix such as collagens type II and aggrecan without inducing chondrocyte hypertrophy or bone-related proteins. BMP-7 also regulates the expression of several other growth factors including insulin-like growth factor-1 (IGF-1) and TGF-β/BMPs (55). A Phase I, multi-center, placebo-controlled RCT showed that IA injections BMP-7 at 0.1 and 0.3 mg showed improvement in WOMAC pain and an increase in the OARSI response rate than placebo, while IA BMP-7 at 1 mg was associated with injection site pain (56). Most adverse events in the BMP-7 group were categorized as mild or moderate, and were similar to the placebo group. Further Phase II trials using the 0.1 and 0.3 mg dose groups are underway. Although BMP-7 has been investigated for several decades in scientific researches, it still requires more clinical trials to evaluate whether it can be used as a clinical treatment for OA.

### Fibroblast Growth Factor-18 (FGF-18)

FGF-18 is involved in chondrogenesis and cartilage repair. In *in vitro* culture of chondrocytes, sprifermin (recombinant human FGF-18) promotes cell proliferation and maintains chondrocyte phonotype, and up-regulates the expression of chondrocyte typical markers (57). A double-blind, placebo-controlled, proof-of-concept RCT with 180 patients was performed to evaluate the safety and effectiveness of IA sprifermin for the treatment of symptomatic knee OA (58). IA sprifermin significantly prevented the thickness and volume loss, and joint space width narrowing of total and lateral femorotibial cartilage in a dose-dependent manner, but cannot reduce cartilage loss in the central medial femorotibial compartment. No severe safety issues were reported related to the study medication. A subsequent pre-specified 3-year analysis of the 5-year Phase II FORWARD study revealed a significant increase in cartilage thickness of femorotibial joint with sprifermin 100 μg treatment compared to placebo at Year 2 and maintained up to Year 3 (59). According to the results obtained so far, sprifermin is effective to increase cartilage thickness of knee OA with acceptable safety profile. The ongoing Phase II trial will further evaluate the safety and effectiveness of this novel OA biologics.

### Human Serum Albumin (HSA)

The low molecular weight fraction of 5% HSA (LMWF-5A) has been reported to produce anti-inflammatory effects by inducing transcriptional changes of mesenchymal stem cell and increasing COX-2, prostaglandin E2 and D2 in inflammation, and up-regulate the transcription of collagen 2α1 during chondrogenic differentiation, thereby being effective in OA treatment (60, 61). Ampion™, commercial LMWF-5A (<5 kDa) developed by Ampio Pharmaceuticals, has been shown to exert anti-inflammatory effects by inhibiting the production of pro-inflammatory cytokines in T-cells (17, 62). A clinical trial of 329 patients with moderate to severe OA under the treatment of Ampion™ over 12 weeks resulted in a significant reduction in WOMAC pain compared to vehicle control (60). Analysis of 3 randomized placebo-controlled trials of 417 patients with severe knee OA (Kellgren-Lawrence grade 4) indicated that Ampion™ significantly improved WOMAC pain, physical function, and patient global assessment scores and higher responder rates compared with saline (63). No severe drug-related deaths or withdrawals associated with adverse events during the HSA treatment have been reported to date and additional long-term clinical trials are required to further determine its clinical benefit.

### β-Nerve Growth Factor (β-NGF) Antibody

Therapies with monoclonal antibodies represent one of the new therapeutic possibilities of many bone diseases including osteoarthritis (64, 65). Tanezumab is an investigational humanized monoclonal antibody against β-NGF, being co-developed by Pfizer and Lilly for treatment of chronic pain in patients with OA. β-NGF has been found with a potential of pain modulation through nociceptor sensitization (66, 67). NGF levels were found elevated in the synovial fluid of OA patients (68). In a clinical trial with 450 patients with knee OA, tanezumab significantly reduced knee pain accompanied with an improved overall assessment (*p* < 0.001) compared to placebo (69). Treatment with tanezumab was associated with a higher rate of adverse events, such as headache, upper respiratory tract infection, and paresthesia, than placebo (68 vs. 55%). Progressively worsening OA was developed in 16 subjects, requiring total joint replacements. However, subsequent studies found that the risk of rapidly progressive OA was greater when tanezumab was used in combination with NSAID than tanezumab alone (70, 71). In June 2017, tanezumab has been granted Fast Track designation by FDA for OA treatment. Therefore, the approval process is expected to be completed relatively quickly.

### Interleukin-1 (IL-1) Inhibitor

IL-1 is involved in OA progression by stimulating the synthesis of proteolytic enzymes, cytokines and other mediators which lead to tissue inflammation and destruction, and by inducing cartilage extracellular matrix degradation (72, 73). Specific inhibition of IL-1 production or activity has been well-documented to prevent the development of experimentally induced OA in animal studies (74, 75). The use of IL-1 receptor inhibitor on OA patients has been reported by two independent RCTs. In one study, 160 patients with symptomatic OA received a single intra-articular injection of anakinra, a recombinant form of IL-1 receptor antagonist, and were evaluated for 12 weeks post-injection (76). In another study, AMG108, an IL-1 receptor antibody, was intra-articularly injected into 159 patients with knee OA once every 4 weeks for 12 weeks (77). Although the tolerability of the IL-1 receptor antagonist/antibody is acceptable, no significant clinical improvement has been reported in either trial compared to placebo control. Although IL-1 inhibitor shows its effectiveness for OA treatment in numerous preclinical studies, its failure to improve OA compared to placebo control in clinical trials warrants further investigation of the mechanisms of action and refinement of treatment regimen.

### Matrix Extracellular Phosphoglycoprotein (MEPE)

OA is often associated with subchondral bone thickening and cartilage hypertrophy. MEPE, a mineral-regulating protein predominantly expressed in osteocytes and odontoblasts, could negatively regulates mineralization and thus show potential for OA treatment (78). TPX-100, a novel 23-amino-acid peptide derived from MEPE and has been used to treat patients with mild to moderate patello-femoral osteoarthritis in 2 phase II clinical trials (NCT01925261, NCT03125499). Significant improvement of KOOS and WOMAC scores was achieved in the TPX-100 treated group compared to placebo controls. However, quantitative MRI revealed no measurable structural modification over 12 months. No serious drug-related adverse events were identified (79). Its safety and effectiveness needs to be validated in long-term clinical trials to further determine its clinical benefit.

### Parathyroid Hormone (PTH)/ Parathyroid Hormone-Related Protein (PTHrP)

PTH or its homolog PTHrP has been reported to inhibit chondrocyte hypertrophy and regulate endochondral ossification in the growth plate through the Ihh-PTHrP regulatory axis (80). It has also been identified that they regulate subchondral bone remodeling and reduce terminal differentiation of articular cartilage (81). Teriparatide, the recombinant human PTH (1-34), has been approved by FDA for treatment of osteoporosis. Systematic administration of teriparatide can effectively inhibit cartilage degradation and abnormal chondrocyte differentiation in injury-induced mouse OA model (82). Combined therapy of IA recombinant human PTHrP (1–40) and collagen-silk scaffold implantation has been shown to significantly reduced chondrocyte terminal differentiation and promote cartilage regeneration in a rabbit osteochondral defect model at 4–6 weeks post-injury (83). Although most of the PTH/PTHrP studies are in the preclinical stage, it's still promising for the future clinical applications. A prospective, Phase II RCT is currently recruiting participants to evaluate teriparatide as a chondroregenerative therapy for OA (NCT03072147). Eighty patients with symptomatic knee OA will be enrolled for a 24-week treatment.

### TGF-β Inhibitor

TGF-β is important to maintain the metabolic homeostasis and structural integrity of articular cartilage. The expression of TGF-β1 was found elevated in the subchondral bone of surgically induced mouse OA model or human OA patients. High levels of active TGF-β1 increased nestin-positive mesenchymal stem cell (MSC) clusters, resulting in abnormal subchondral bone formation with increased angiogenesis. Knockout of the TGF-β type II receptor (TβRII) in nestin-positive MSCs inhibited the development of OA in experimentally induced OA mouse model. Furthermore, inhibition of TGF-β1 signaling by injection of a TGF-β type I receptor inhibitor (SB-505124) or by implantation of an antibody to TGF-β (1D11) in alginate beads in subchondral bone protected against OA degeneration (84). The TGF-β inhibitor is a new target for future drug development in OA treatment.

### Cholesterol Metabolism and Inverse Agonist of Retinoic Acid-Related Orphan Receptor Alpha (RORα)

The interaction between cholesterol metabolism and ROR plays an important role in the pathogenesis of OA, and the CH25H-CYP7B1-RORα axis of cholesterol metabolism has cartilage specificity. RORα is a downstream target of CH25H-CYP7B1 cholesterol metabolism axis, which can be activated by cholesterol and its oxysterol metabolite, thereby up-regulating the expression of MMP3, MMP13, and ADAMTS5, causing cartilage destruction of OA. A study showed that, double knockout of INSIG1 and INSIG2 stimulates cholesterol synthesis in chondrocytes to increase the severity of mice OA (85). A very recent study showed that high cholesterol diet resulted in elevated serum cholesterol levels and increased severity of OA compared to conventional diet in experimental OA of mice (86). Moreover, intra-articular injection of SR3335, an inverse agonist of RORα significantly reduced cartilage destruction caused by adenoviruses-mediated overexpressing of RORα or medial meniscus surgery, and inhibited the up-regulation of MMP3 and MMP13 caused by cholesterol and its metabolites (86). Therefore, cholesterol metabolism and RORα might be novel therapeutic targets for OA.

## Conclusion

Despite currently available therapies and ongoing research, there's still no “gold standard” or “magic bullet” in the treatment of OA. Physical measures, traditional drugs, and even surgery remain the first line treatments (for different OA types) in today's clinical practice. The desired treatments should not only aim to alleviate pain and symptoms but also help rebuild normal cartilage structure and restore joint function. Considering that OA is a disease of the whole joint rather than the articular cartilage itself, the search of novel therapeutic targets in the process of subchondral bone remodeling or synovial inflammation is necessary. Traditional OA drugs have been confirmed to be effective in pain and inflammation control, but cannot reverse the process underlying OA. Frequently-occurred side effects hinder their widespread use. Compared with traditional OA drugs, emerging disease-modifying OA drugs which act by reducing inflammation, enhancing cartilage repair, and inhibiting OA degeneration, are associated with more pronounced effects and fewer side effects than traditional medications. However, most of the emerging drugs are in the preclinical phase. Long-term RCTs are warranted to better elucidate the safety and effectiveness of the emerging medications for OA treatment.

## Author Contributions

WZ and WC performed the research and wrote the article. JX and WR designed this research. JX, WR, and JZ provided supervision and revised the paper.

### Conflict of Interest Statement

The authors declare that the research was conducted in the absence of any commercial or financial relationships that could be construed as a potential conflict of interest.
